# Why do mentally ill, homeless people use substances?

**DOI:** 10.1192/j.eurpsy.2021.628

**Published:** 2021-08-13

**Authors:** L. Elmquist, M. Henriksen, J. Nordgaard

**Affiliations:** 1 Mental Health Center Amager, Mental health Services in The Capital Region of Denmark, Copenhagen V, Denmark; 2 Mental Health Center Amager, Mental Health Services in the Capital Region of Denmark, Copenhagen V, Denmark; 3 Center For Subjectivity Research, University of Copenhagen, Copenhagen, Denmark; 4 Department Of Clinical Medicine, University of Copenhagen, Copenhagen, Denmark

**Keywords:** Homelessness, dual diagnosis, Substance use

## Abstract

**Introduction:**

In the Danish social welfare system, few people are homeless solely for economic reasons. In fact, 38% of homeless people suffer from both substance use and a psychiatric disorder, making diagnostic assessment and treatment difficult. This patient group, with dual diagnoses, often fail to receive effective treatment, and the consequences are far reaching and detrimental. A more comprehensive grasp of the history and patterns of substance use in these patients may contribute to improve their treatment.

**Objectives:**

To identify the role and patterns of substance use in mentally ill, homeless people.

**Methods:**

50 homeless, mentally ill patients are examined in comprehensive interviews, exploring the relationship between substance use, homelessness, and suffering from a mental disorder. The data are analyzed quantitatively as well as qualitatively using thematic analysis.

**Results:**

Preliminary results indicate that substance use in mentally ill homeless patients is a complex phenomenon. On the one hand, substance use seems to contribute to keep the patient homeless and makes it difficult for the patient to get the necessary psychiatric help. On the other hand, substance use also appear to play an important part in coping with life on the streets by offering some kind of social contact and some relief from a desperate situation.

**Conclusions:**

It seems that the triad of substance use, mental illness, and homelessness somehow reinforce each other and simultaneously locks the situation. New approaches for disentangling this locked situation and avoiding this ‘Bermuda triangle’ is needed.

